# Supplementary material for: Characteristics and Residual Health Risk of Organochlorine Pesticides in Fresh Vegetables in the Suburb of Changchun, Northeast China
Source: Int J Environ Res Public Health. 2022 Oct 1;19(19):12547. doi: 10.3390/ijerph191912547 (PMC9566688; doi:10.3390/ijerph191912547)
Supplement: Supplementary file 1 [file ijerph-19-12547-s001.zip › ijerph-1917984-supplementary.pdf]

Characteristics and Residual Health Risk of Organochlorine Pesticides in Fresh Vegetables in the Suburb of Changchun, Northeast China

Nan Wang [1], Zhengwu Cui [1,2,*], Yang Wang [1,*] and Jingjing Zhang [3]

[1] Northeast Institute of Geography and Agroecology, Chinese Academy of Sciences, Changchun 130102, China
[2] University of Chinese Academy of Sciences, Beijing 100049, China
[3] College of Resources and Environment, Henan Agricultural University, Zhengzhou 450002, China
* Correspondence: cuizhengwu@iga.ac.cn (Z.C.); wangyangw@iga.ac.cn (Y.W.)

Supplementary Information

Table S1. Percentage of combined pollution of organochlorine pesticides in edible parts of vegetables from Changchun, China.

Vegetables	No. of samples analyzed	No. of samples with detectable residues (%）	No. of samples ≤MRL (%)	No. of samples > MRL (%)
Chinese cabbage	28	60.71	46.43	14.29
Welsh onion	40	77.50	55.00	22.50
Radish	16	62.50	43.75	18.75
Cucumber	31	51.61	48.39	3.23
Pepper	29	48.28	41.38	6.90
Eggplant	34	41.18	38.24	2.94
Tomato	36	47.22	44.44	2.78
Leafy vegetables[a]	68	70.59	51.47	19.12
Root vegetables[b]	16	62.50	43.75	18.75
Fruit vegetables[c]	130	46.92	43.08	3.85
Total	214	55.61	45.79	9.81

[a] Included Chinese cabbage (*Brassica campestris* L. ssp. *pekinensis*(Lour.) Olsson) and Welsh onion (*Allium fistulosum* L. var. *giganteum* Makino).

[b] Included radish (*Raphanus sativus* L.).

[c] Included cucumber (*Cucumis staivus* L.), pepper (*Capsicum annuum* L.), eggplant (*Solanum melongena* L.) and tomato (*Lycopersicon* esculentum Mill).

Table S2. Organochlorine pesticides detected in edible parts of vegetables from Changchun, China.

OCPs	MRL/ng·g^{-1}	Leafy vegetables (n=68)				Root vegetables (n=16)		Fruit vegetables (n=130)								Total(n=214)	
		Chinese cabbage (n=28)		Welsh onion (n=40)		Radish (n=16)		Cucumber (n=31)		Pepper (n=29)		Eggplant (n=34)		Tomato (n=36)			
		D.R/%	>MRL/%	D.R/%	>MRL/%	D.R/%	>MRL/%	D.R/%	>MRL/%	D.R/%	>MRL/%	D.R/%	>MRL/%	D.R/%	>MRL/%	D.R/%	>MRL/%
α-HCH	/	25.00	/	30.00	/	18.75	/	19.35	/	20.69	/	11.76	/	16.67	/	20.56	/
β-HCH	/	32.14	/	37.50	/	25.00	/	25.81	/	24.14	/	23.52	/	22.22	/	27.57	/
γ-HCH	/	35.71	/	35.00	/	18.75	/	25.81	/	20.69	/	26.47	/	27.78	/	28.04	/
δ-HCH	/	14.29	/	7.50	/	12.50	/	16.13	/	10.34	/	14.71	/	11.11	/	12.15	/
∑HCHs	50	35.71	3.57	37.50	7.50	31.25	6.25	32.25	0	24.14	3.44	26.47	0	27.78	0	30.84	2.80
o,p′-DDT	/	25.00	/	27.50	/	31.25	/	29.03	/	20.69	/	20.59	/	19.44	/	24.30	/
p,p′-DDT	/	28.57	/	35.00	/	31.25	/	22.58	/	20.69	/	20.59	/	16.67	/	24.77	/
p,p′-DDD	/	28.57	/	35.00	/	25.00	/	16.13	/	13.79	/	17.65	/	8.33	/	20.56	/
p,p′-DDE	/	21.43	/	32.50	/	25.00	/	19.35	/	17.24	/	23.52	/	13.89	/	22.00	/
∑DDTs	50	28.57	7.14	37.50	10.00	31.25	6.25	29.03	0	20.69	3.44	26.47	0	19.44	0	27.57	3.74
cis-chlordane	/	17.86	/	25.00	/	18.75	/	22.58	/	20.69	/	14.71	/	16.67	/	19.62	
trans-chlordane	/	14.29	/	22.50	/	18.75	/	19.35	/	17.24	/	11.76	/	11.11	/	16.36	
∑Chlordans	20	17.86	7.14	25.00	7.50	18.75	6.25	22.58	3.23	20.69	0	14.71	2.94	16.67	2.77	19.63	4.21
Heptachlor	20	14.29	0	12.50	0	12.50	0	16.13	0	13.79	0	11.76	0	11.11	0	13.08	0

ΣHCHs=α-HCH+β-HCH+γ-HCH+δ-HCH; ΣDDTs= o,p′-DDT+ p,p′-DDT+p,p′-DDD+p,p′-DDE+; ΣChlordane=cis-chlordane+trans-chlordane

MRL: Maximum Residue Limit was obtained by GB 2763-2021, China.
